# Subcutaneous Interferon β-1a May Protect against Cognitive Impairment in Patients with Relapsing–Remitting Multiple Sclerosis: 5-Year Follow-up of the COGIMUS Study

**DOI:** 10.1371/journal.pone.0074111

**Published:** 2013-08-30

**Authors:** Francesco Patti, Vincenzo Brescia Morra, Maria Pia Amato, Maria Trojano, Stefano Bastianello, Maria Rosalia Tola, Salvatore Cottone, Andrea Plant, Orietta Picconi, COGIMUS Study Group

**Affiliations:** 1 Department of Neurology, Multiple Sclerosis Centre Sicilia Region, First Neurology Clinic, University Hospital, Catania, Italy; 2 Department of Neurological Science, University of Naples Federico II, Naples, Italy; 3 Department of Neurology, University of Florence, Florence, Italy; 4 Department of Neurological and Psychiatric Sciences, University of Bari, Piazza Giulio Cesare, Bari, Italy; 5 Neuroradiological Department, University of Pavia, Corso Strada Nuova, Pavia, Italy; 6 U.O. Neurology, Department of Neuroscience and Rehabilitation, Azienda Università-Ospedale S. Anna, Ferrara, Italy; 7 Centro Di Riferimento Regionale di Neuroimmunologia, Via Ninni Cassarà, Palermo, Italy; 8 Caudex Medical, Seacourt Tower, West Way, Oxford, United Kingdom; 9 Opera CRO srl, Genova, Italy; Institute Biomedical Research August Pi Sunyer (IDIBAPS) - Hospital Clinic of Barcelona, Spain

## Abstract

**Objective:**

To assess the effects of subcutaneous (sc) interferon (IFN) -1a on cognition over 5 years in mildly disabled patients with relapsing–remitting multiple sclerosis (RRMS).

**Methods:**

Patients aged 18–50 years with RRMS (Expanded Disability Status Scale score ≤4.0) who had completed the 3-year COGIMUS study underwent standardized magnetic resonance imaging, neurological examination, and neuropsychological testing at years 4 and 5. Predictors of cognitive impairment at year 5 were identified using multivariate analysis.

**Results:**

Of 331 patients who completed the 3-year COGIMUS study, 265 participated in the 2-year extension study, 201 of whom (75.8%; sc IFN β-1a three times weekly: 44 µg, n = 108; 22 µg, n = 93) completed 5 years' follow-up. The proportion of patients with cognitive impairment in the study population overall remained stable between baseline (18.0%) and year 5 (22.6%). The proportion of patients with cognitive impairment also remained stable in both treatment groups between baseline and year 5, and between year 3 and year 5. However, a significantly higher proportion of men than women had cognitive impairment at year 5 (26.5% vs 14.4%, p = 0.046). Treatment with the 22 versus 44 µg dose was predictive of cognitive impairment at year 5 (hazard ratio 0.68; 95% confidence interval 0.48–0.97).

**Conclusions:**

This study suggests that sc IFN β-1a dose-dependently stabilizes or delays cognitive impairment over a 5-year period in most patients with mild RRMS. Women seem to be more protected against developing cognitive impairment, which may indicate greater response to therapy or the inherently better prognosis associated with female sex in MS.

## Introduction

Cognitive impairment is an important feature of multiple sclerosis (MS), affecting up to 65% of patients [1]. Cognitive symptoms may develop from the early stages of MS, sometimes as the presenting symptoms, and in any form of the disease (clinically isolated syndrome [CIS], relapsing–remitting MS [RRMS], or primary or secondary progressive MS) [2]. Once present, cognitive symptoms are unlikely to resolve and the level of impairment is believed to increase with worsening of physical disability [3], disease duration [4], [5], and the onset of progressive disease [4], [5]. Deficits in memory, learning, attention, and information-processing ability, most commonly observed in MS, may reflect damage to specific brain regions that do not affect physical functioning. Therefore, cognitive decline can indicate disease progression in patients with stable physical function [5], [6].

Cognitive symptoms alone can negatively affect many aspects of patients' daily lives, including employment and social relationships, reducing overall quality of life [7], [8]. In addition, common MS comorbidities, such as fatigue and depression, can impair cognitive function and further increase disability levels [4], [9], [10].

Despite its high prevalence in MS, cognitive impairment is rarely measured as part of standard clinical assessments because many cognitive tests require specialist training and must be administered by a certified neuropsychologist. In addition, tests are often time consuming to perform [2]. For patients with cognitive impairment, treatment is based on symptomatic therapies that aim to optimize remaining cognitive function and thus reduce the impact of cognitive decline [11], [12]. Alternatively, pharmacological treatment of comorbidities affecting cognitive performance can provide benefits for patients, for example acetylcholinesterase inhibitors, which are widely used to treat Alzheimer's disease, may also benefit patients with MS [13].

There is considerable evidence to indicate that disease-modifying drugs (DMDs) can significantly improve outcomes for patients with MS by reducing lesion development and improving clinical measures of disease, such as relapse rate [14]. The observation that some magnetic resonance imaging (MRI) disease measures, such as lesion load and brain volume, correlate with cognitive impairment suggests that DMD treatment may also prevent or delay cognitive decline by attenuating inflammatory processes and preventing the development of new brain lesions or progressive brain atrophy [12], [13]. However, as the pivotal trials of DMDs did not, in general, include cognitive assessments, the cognitive benefits of DMDs in patients with MS are unconfirmed.

The COGIMUS (COGnitive Impairment in MUltiple Sclerosis) study evaluated cognitive decline in mildly disabled Italian patients with RRMS receiving treatment with interferon (IFN) β-1a, 22 or 44 µg (Rebif®; Merck Serono S.A., Switzerland), administered subcutaneously (sc) three times weekly (tiw). In this study, cognitive impairment was assessed using the Rao's Brief Repeatable Battery (BRB) and the Stroop Color–Word Task (Stroop Test), which have been validated for use in patients with MS and for which Italian normative values are available [15]. After 3 years' follow-up, it was found that sc IFN β-1a may have dose-dependent cognitive benefits in this patient group. At year 3, the proportion of patients with cognitive impairment was significantly higher in the 22 µg group than in the 44 µg group (p = 0.03) and the risk of cognitive impairment was reduced by 32% with the 44 µg dose [16]. These findings may further support early initiation of high-dose IFN β-1a treatment in patients with RRMS. Here we report clinical and cognitive outcomes from the 2-year extension of the study, giving a total of 5 years' follow-up.

## Methods

COGIMUS was a prospective, 3-year, multicenter, observational, Italian cohort trial. Patients were enrolled between September 2003 and March 2005. Methodological details have been reported elsewhere [16]. Following completion of the study, patients were eligible to enter a 2-year extension study, with a total follow-up of 5 years.

### Patients

Eligibility criteria have been previously described [17]. Briefly, patients were aged 18–50 years with a diagnosis of RRMS (McDonald criteria), had an Expanded Disability Status Scale (EDSS) score of ≤4.0 and were naïve to DMD treatment. All patients at participating centers who had completed the core 3-year study were invited to participate in the 2-year extension study. All patients gave written informed consent prior to undergoing any assessments not performed as part of their routine care. The study protocol was first approved in September 2003 by the ethics committee of Policlinico, University of Catania, and then in the following 9 months by the local ethics committees of: Garibaldi Hospital (Catania), San Camillo Hospital (Rome), University Federico II of Naples, Cardarelli Hospital (Naples), Careggi Hospital (Florence), Orbassano Hospital (Piedmont Region), Palermo – ASP 6 (Sicily Region), University Tor Vergata (Rome), University of Palermo, Prato Hospital (Tuscany Region), University of Padova, University of Udine, Macerata Hospital (Marche Region), Giglio–San Raffaele Hospital, ASP 7 (Sicily Region), University of Chieti, University of Novara, Fidenza Hospital (Emilia Romagna Region), Ascoli Piceno Hospital (Marche Region), University of Ancona, University of Ferrara, University of Bari, Sant'Antonio Abate Hospital (Gallarate, Lombardy Region), Arezzo Hospital (Tuscany Region), University of Trieste, State University of Milan, University of Torino, La Spezia Hospital (Liguria Region), University of L'Aquila, and Avellino Hospital (Campania Region). The study was conducted in accordance with the principles expressed in the Declaration of Helsinki.

### Treatment

In the core study, patients were assigned to IFN-β treatment, with the formulation and dose at the discretion of their treating physician [16], [17]. Of those who received sc IFN β-1a (N = 459), 223 (48.6%) received the 22 µg dose and 236 (51.4%) received the 44 µg dose. All patients who completed the 5-year follow-up continued on the same treatment as at year 3 for the duration of the extension study. Relapses were treated with corticosteroids, and flu-like symptoms with non-steroidal anti-inflammatory drugs or paracetamol. DMDs other than the study drug were not permitted.

### Study objectives and endpoints

The primary objective of the extension study was to determine the effects of two doses of sc IFN β-1a on cognition over 5 years; the primary endpoint was the proportion of patients with cognitive impairment at year 5. The main secondary objective was to identify factors that predicted the presence of cognitive impairment after 5 years on study. In addition, discontinuations and reasons for treatment discontinuation, and adverse events (AEs) during years 3–5, were recorded. Patients who discontinued treatment were followed regularly in the clinical trial setting and were included in the analyses if they had cognitive assessments at all time points, regardless of whether they had discontinued treatment.

### Evaluation of disease status

Clinical and MRI assessments during the core study have been reported previously [17]. Patients attended two further visits at years 4 and 5 that comprised neurological assessment, including EDSS score, recording of relapses, and MRI (25 of 34 centers to year 3, and 19 centers from years 3 to 5).

### Neuropsychological evaluation

All patients underwent neuropsychological evaluation at baseline and every 12 months during the core study. Two further neuropsychological assessments were performed at years 4 and 5, as described previously [17], namely the BRB (alternate versions administered in the order A, B, A, B) and the Stroop Test. Cognitive impairment was defined as 1 standard deviation (SD) below the mean Italian normative values for each cognitive test [15]. Cognitive testing of patients who had an ongoing relapse at the time of the scheduled assessment was delayed until 30 days after the last steroid injection.

### Statistical analyses

For outcome measures at 5 years, only patients with 5 years of follow-up were included in the analyses. No imputation of missing data was considered. Analyses at 5 years were exploratory without adjustment for multiplicity. Cognitive data from baseline and years 1, 3, and 5 only (BRB version A) were analyzed to avoid differences due to administration of alternate versions of the BRB. The following tests were conducted: Pearson chi-square and McNemar tests to compare categorized proportions, Cox proportional hazards regression to compare longitudinal data on cognitive impairment, Cochran test for *k*-related samples to assess variation over time in the percentage of patients with cognitive impairment, and Friedman test for *k*-related samples to assess variation over time in the number of impaired tests in the study population and each treatment group. In addition, Kaplan–Meier survival curves were constructed to evaluate longitudinal differences between treatments. Risk factors for the presence of cognitive impairment over 5 years were identified using a multivariate regression model, which was developed by sequentially adding variables with a significant hazard ratio in univariate analyses. Statistical significance was set at 0.05.

## Results

### Patients and baseline characteristics

Of the 40 original participating centers, 23 (accounting for 80.1% [265/331] of patients from the 3-year follow-up cohort) participated in the extension study. Of the 265 eligible patients, 201 (75.8%; sc IFN β-1a tiw: 44 µg, n = 108; 22 µg, n = 93) completed the 5-year follow-up and were included in these analyses. The mean duration of follow-up was 5.6 years (range 4.5–6.1 years). Mean (SD) age was 39 (8.2) years and mean (SD) disease duration was 8 (4.4) years (mean [SD] disease duration at baseline: 3.9 [4.4 years]). No differences were found between patients who did or did not participate in the 5-year follow-up in terms of baseline clinical and demographic characteristics, neuropsychological performance, or proportions receiving the 44 or 22 µg dose, with the exception of mean (SD) Environmental Status Scale score, which was greater in patients who had the 5-year follow-up (1.63 [2.5]) than those who did not (1.29 [2.4]). The male:female ratio was 0.6. Overall, there was no difference in the proportion of patients with or without cognitive impairment at year 3 (the end of the core study) who went on to participate in the 2-year extension study and complete the 5-year follow-up (Pearson chi-squared test  = 0.574; Table 1).

**Table 1:** Proportion of patients with and without cognitive impairment at year 3 (end of the core study) who did/did not complete follow-up at year 5.

Cognitive status at year 3 (n = 265)	Completed 5-year follow-up,%			
	No (n = 64)	Yes (n = 201)	Total	Pearson chi-squared test
Not impaired (n = 216)	79.49	81.92	80.78	0.574
Impaired (n = 49)	20.51	18.08	19.22	
Total	100.0	100.0	100.0	

Between years 3 and 5, 64 patients discontinued treatment. Discontinuation rates were similar in both IFN β-1a groups: 35 patients receiving 44 µg and 29 patients receiving 22 µg. Reasons for discontinuation were: AEs, 5 (7.8%) patients; lost to follow-up, 28 (43.8%); lack of efficacy, 11 (17.2%); pregnancy/planning to conceive, 10 (15.6%); other, 10 (15.6%). ‘Other’ reasons were predominantly subjective, such as ‘patient decided not to continue’. There were no discontinuations due to injection-site reactions.

### Cognitive impairment at 5 years

**Figure 1:**
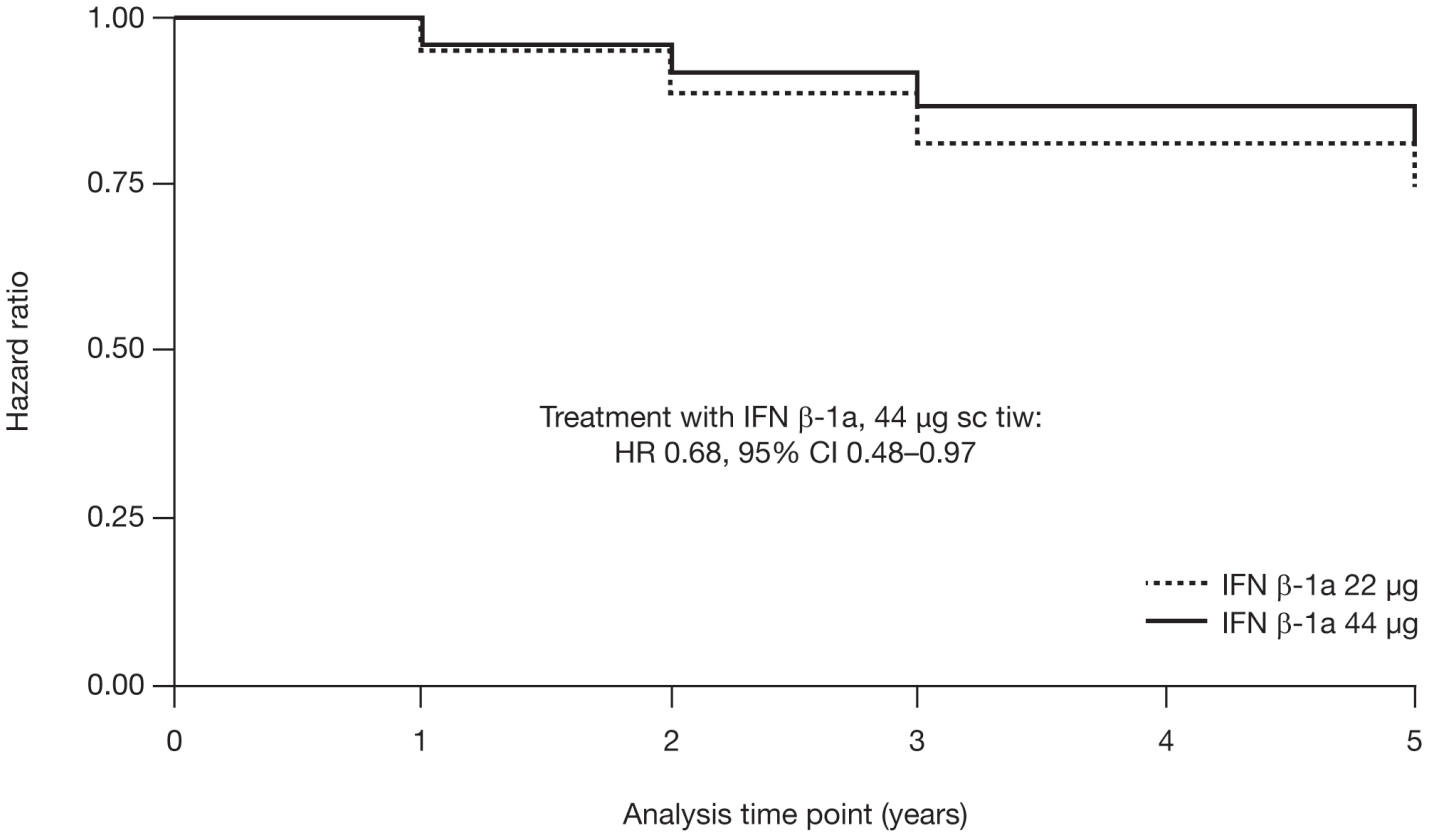
Kaplan–Meier survival curve for patients without cognitive impairment at baseline. Development of cognitive impairment (i.e. impaired performance on ≥3 cognitive tests) over 5 years in patients receiving IFN β-1a treatment (22 or 44 µg sc tiw). Cl, confidence interval; HR, hazard ratio; IFN, interferon; sc, subcutaneously; tiw, three times weekly.

**Figure 2:**
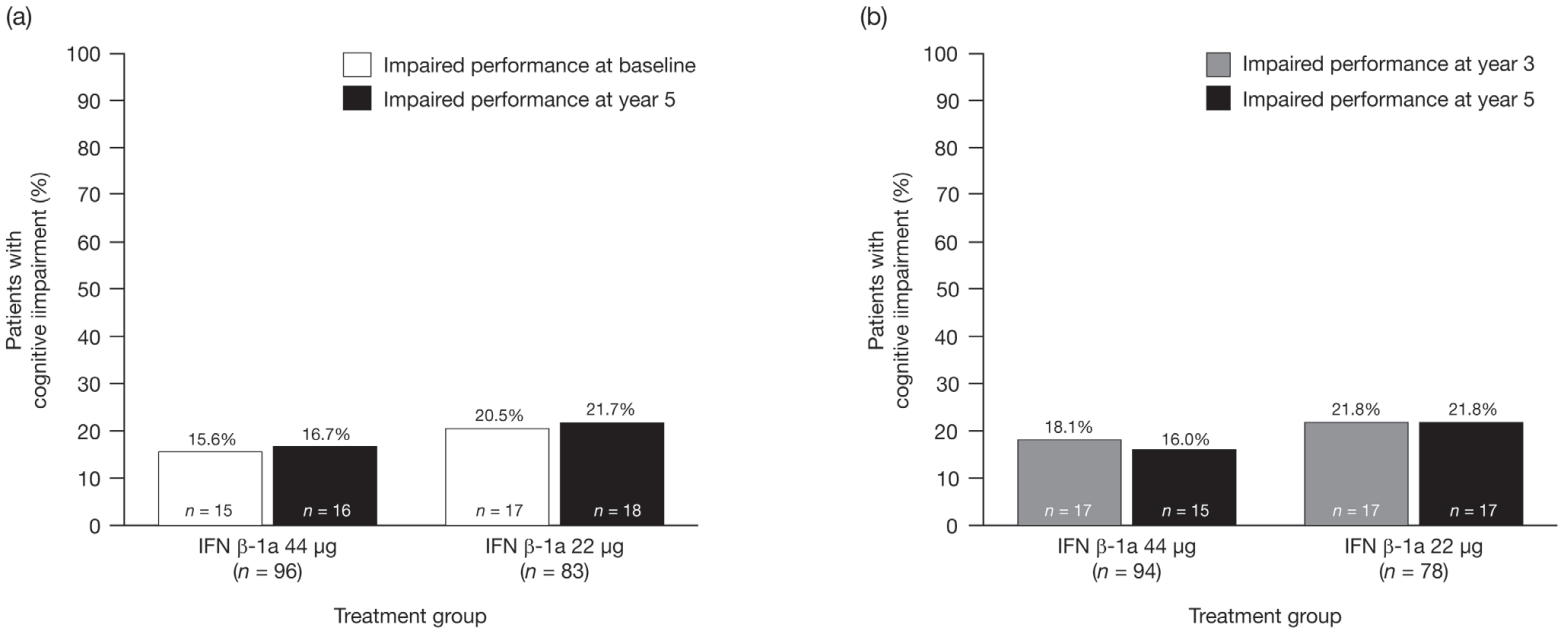
Cognitive impairment at (a) baseline and year 5, and (b) years 3 and 5. Cognitive impairment defined as impaired performance on ≥3 cognitive tests. IFN, interferon.

A Cox proportional hazards survival analysis was performed to assess the development of cognitive impairment (proportion of patients with ≥3 impaired cognitive tests) during the 5-year study. Figure 1 shows Kaplan-Meier survival curves for this analysis, by treatment (discussed further below). The overall proportion of patients with cognitive impairment did not increase significantly over the 5-year period. Among patients with data available at all time points, the proportion with cognitive impairment was 18.0% at baseline and 22.6% at year 5 (Cochran test  = 0.392; Table 2). Similarly, only small and non-significant increases were seen in the proportion of patients with cognitive impairment in each treatment group. In the 44 µg group, 15.6% of patients at baseline and 16.7% at year 5 had cognitive impairment; in the 22 µg group, the corresponding proportions were 20.5% and 21.7% (Figure 2a). The proportion of patients with cognitive impairment also remained stable between 3 and 5 years' follow-up in both the 44 µg group (18.1% vs 16.0%, respectively) and 22 µg group (21.8% vs 21.8%, respectively; Figure 2b). The mean (SD) number of cognitive tests of impairment did not differ significantly over time, neither in the entire study cohort nor in either treatment group, except for baseline versus year 1 in the 22 µg group (1.4 [1.7] vs 1.8 [2.0]; p = 0.020).

**Table 2:** Proportion of patients with and without cognitive impairment^a^ at baseline and years 1, 3 (end of core study), and 5 (end of extension study).

	Baseline		Year 1		Year 3		Year 5	
IFN β-1a sc tiw group	With impairment, *n* (%)	Without impairment, *n* (%)	With impairment, *n* (%)	Without impairment, *n* (%)	With impairment, *n* (%)	Without impairment, *n* (%)	With impairment, *n* (%)	Without impairment, *n* (%)
44 µg (n = 69)	10 (14.5)	59 (85.5)	14 (20.3)	55 (79.7)	12 (17.4)	57 (82.6)	14 (20.3)	55 (79.7)
22 µg (n = 64)	14 (21.9)	50 (78.1)	16 (25.0)	48 (75.0)	13 (20.3)	51 (79.7)	16 (25.0)	48 (75.0)
All patients (n = 133)	24 (18.0)	109 (82.0)	30 (22.6)	103 (77.4)	25 (18.8)	108 (81.2)	30 (22.6)	103 (77.4)

a Defined as impaired performance on ≥3 tests of the Rao's Brief Repeatable Battery and Stroop Color–Word Task.

Cochran test  = 0.392.

IFN =  interferon; sc =  subcutaneously; tiw =  three times weekly.

### Predictors of cognitive impairment at year 5

Kaplan-Meier survival curves confirmed the benefits of receiving the higher versus the lower dose of sc IFN β-1a treatment on cognitive impairment during the 5-year study (Figure 1). The hazard ratio (95% confidence interval) for the 44 µg versus 22 µg dose was 0.68 (0.48–0.97) over 5 years.

**Figure 3:**
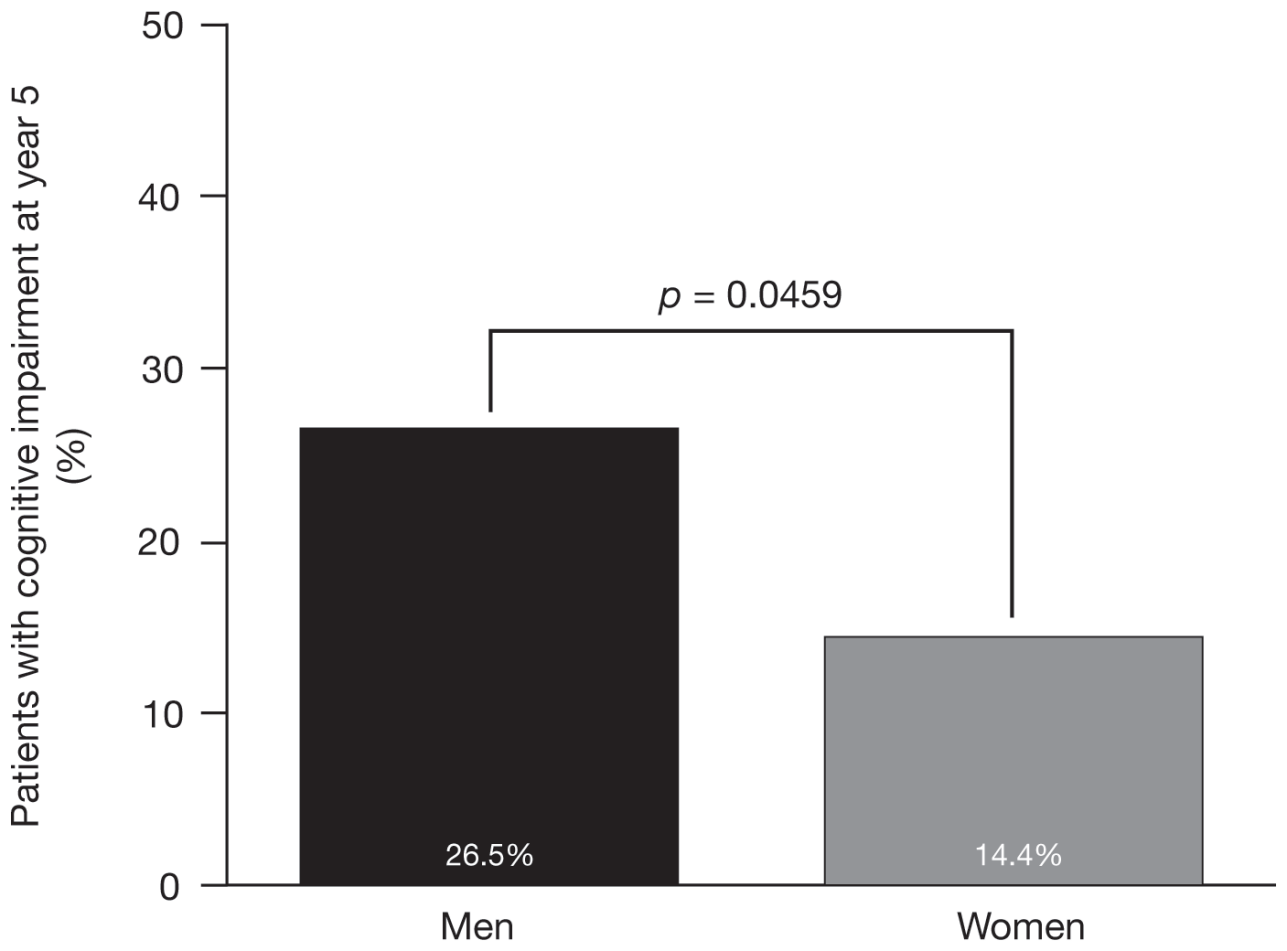
Proportion of men and women with cognitive impairment at year 5. Cognitive impairment defined as impaired performance on ≥3 cognitive tests.

At year 5, the proportion of men with cognitive impairment was significantly higher than the proportion of women (26.5% [18/68] vs 14.4% [16/111], p = 0.0459; Figure 3).

### Clinical outcomes

Over the 5-year period, the mean relapse rate per patient per year was 0.21. The mean relapse rate remained stable between years 3 and 5. Median EDSS scores also remained stable between years 3 and 5 (median score at year 5: 2.0; interquartile range 2). The proportion of patients who were free from disability progression (as assessed by EDSS score) was 84% at year 3 and 71% at year 5. At the 5-year follow-up, 82% of patients who had been progression-free at year 3 had an unchanged level of physical disability. The proportion of patients who were free from disease progression at year 5 was similar in those with and without cognitive impairment at year 5 (33% vs 27%, respectively). There were no differences in clinical outcomes between treatment groups.

### Safety

AEs were consistent with the known safety profile of IFN β-1a [18]. The most common AEs reported over the 5-year follow-up were injection-site reactions (30% of patients), flu-like symptoms (15% of patients), and depression (2% of patients). Overall, 50% of AEs were classified as mild in severity.

## Discussion

In this extension study, we found that, in the study population as a whole, the proportion of patients with impaired cognitive function remained stable over the 5 years of follow-up. However, after 5 years of treatment with IFN β-1a, a higher percentage of men than women had cognitive impairment. These results suggest that sc IFN β-1a may have a protective effect on cognitive performance and that this effect may be greater in women than in men.

The current finding that the level of cognitive impairment remained stable during the 5 years confirms and extends our previous observations [16], suggesting that sc IFN β-1a may stabilize cognitive function in mildly disabled patients with MS. Natural history studies of cognitive impairment in patients with MS indicate that cognitive performance would be expected to decline by approximately 5% per year in this patient group [6]. Indeed, the proportion of patients with cognitive impairment has been reported to increase substantially from 29% to 54% during the first 5 years after a CIS [19], and from 52.3% to 71.4% in the first 7 years, after diagnosis of MS [8]. Significant cognitive deterioration over 5 years in patients with CIS or MS with a disease duration of ≤6 years has also been reported, particularly in the domains of working memory, speed of information processing, and spatial memory [20]. In contrast, we found no significant increase in the proportion of patients with cognitive impairment over the same timeframe in the 5-year follow-up cohort (mean [SD] duration of disease at baseline: 3.9 [4.4 years]). Considering that cognitive function would be expected to deteriorate over 5 years in the absence of treatment, including in patients with intact cognitive function at baseline [21], the present results confirm our previous findings suggesting that sc IFN β-1a can prevent or delay the onset of cognitive symptoms in patients with MS [16]. Our observation of a dose effect, with the 44 µg dose of sc IFN β-1a being a significant predictor of absence of cognitive impairment at year 5, provides further evidence of a beneficial treatment effect.

How IFN β-1a treatment may bring about cognitive benefits is an interesting topic for debate. A likely explanation is that cognitive effects are a result of the known anti-inflammatory actions of IFN β, which reduce lesion development in the central nervous system (CNS). Correlations between cognitive function and MRI measures of disease have been reported [2], thus supporting this theory. There is increasing evidence to indicate that MS-related changes in cortical matter (lesions and atrophy) play a major role in the development of cognitive symptoms [22]. Recently, sc IFN β-1a has been shown to significantly decrease the development of new cortical lesions and cortical atrophy, which could further explain how sc IFN β-1a protects against cognitive decline in patients with MS [23].

In addition to its immunomodulatory properties, IFN β may also indirectly protect against neuronal damage or promote repair by increasing the production of neurotrophic factors, including nerve growth factor and brain-derived neurotrophic factor [24], [25]. However, the relationship between neurotrophic factor production and cognitive outcomes in patients with MS has not been studied.

Our observation that a greater proportion of men than women had cognitive impairment at year 5 is intriguing, particularly as this difference was not observed at the end of the 3-year core study, and suggests a better response to sc IFN β-1a in women, at least for this outcome. As in other autoimmune diseases, sex differences have been reported in MS susceptibility [26]–[28]. Differences in disease course and severity have also been highlighted, with male sex being associated with a more progressive form of the disease and worse outcomes [28], [29]. Indeed, large epidemiological studies have shown that men reach the same level of disability (EDSS score) as women in a shorter time from diagnosis, are more likely to present with a primary progressive course, and are more susceptible to destructive lesions than women; in contrast, inflammatory lesions seem to be more prevalent in women [30], [31].

The underlying causes of these sex differences are unknown; however, genetic predisposition between sexes [32], [33], the modulation of immune responses by sex hormones, inflammatory processes, tissue injury and repair mechanisms, and possible neuroprotective effects seem to play a part [34], [35]. Consistent with a role for sex hormones is the observation that relapse rates decrease during pregnancy and increase post partum [36], [37]. Our current findings are in agreement with previous studies showing a differential response to IFN β in women and men with MS regarding disability progression [38], although another study in patients with RRMS did not find sex differences regarding response to intramuscular IFN β-1a [39]. It is also possible that the apparent lower response to IFN β-1a in men seen here is, in fact, simply due to the inherently worse prognosis in men; that is, in the absence of treatment, the degree of cognitive decline in men may have been even greater than was observed.

COGIMUS confirmed that cognitive impairment affects a significant proportion of patients from the early stages of MS: over half of all patients in this cohort had impaired performance on at least one cognitive test despite being at an early stage of the disease and having a mild level of disability at study entry [16]. As existing cognitive impairment is a risk factor for further cognitive decline [40], it is clearly important that patients with cognitive symptoms are identified and their treatment tailored as necessary. However, the observation that cognitive impairment can develop during the first 5 years of the disease in patients who previously had no evidence of cognitive symptoms highlights that all patients are potentially at risk of cognitive impairment [19]. The importance of initiating early DMD treatment to prevent or slow the accumulation of damage to the CNS, including brain atrophy [41], and thus physical disability, is now recognized. As cognitive decline occurs in the absence of ongoing relapse or disability progression even in the early stages of disease [21], our findings suggest that early IFN-β treatment may not only protect those with cognitive symptoms from further cognitive decline, but may also prevent the development of cognitive impairment. Whether these observations reflect the prevention of damage to cortical tissue would be an interesting topic for further investigation.

Limitations of this study should be considered when interpreting these findings. The lack of an untreated control group and study discontinuation rate of over 20% between year 3 and completion of the 2-year extension limit the conclusions that can be drawn regarding efficacy. Several centers from the original trial did not participate in the extension phase, accounting for a considerable proportion of the reduction in patient numbers between baseline and year 5. There may also have been a selection bias for patients who are doing well on treatment; patients with declining cognitive function may have been more likely to drop out. However, as there was no significant difference between the proportion of patients with or without cognitive impairment at year 3 who participated in the extension study, cognitive impairment at the end of the core study did not appear to be a predictor of lack of participation at year 5. One limitation in this respect is the lack of availability of patient data at year 4. Furthermore, because cognitive performance was evaluated using a different version of the BRB (version B) in year 2 from that used in years 1, 3, and 5 (version A), and the two versions differ slightly regarding the weight given to some cognitive functions, year 2 data were excluded from the analysis to ensure that the longitudinal data were comparable. Finally, the differential effects in men and women could have been influenced by the different numbers of men and women, or by other possible differences between the sexes, such as adherence to treatment, which were not assessed.

Despite the limitations, the results reported here add to the evidence suggesting that sc IFN β-1a may have dose-dependent cognitive benefits in patients with RRMS. Here we also demonstrate that these benefits persist over at least 5 years of treatment and may be more pronounced in women than in men, although it is possible that the sex difference reflects inherently poorer prognosis in men. Additionally, sc IFN β-1a was shown to achieve good disease control and was well tolerated. Our results further support the clinical benefit of initiating sc IFN β-1a treatment, even in patients with mild physical disability.
